# Individual Differences in Motor Noise and Adaptation Rate Are Optimally Related

**DOI:** 10.1523/ENEURO.0170-18.2018

**Published:** 2018-07-31

**Authors:** Rick van der Vliet, Maarten A. Frens, Linda de Vreede, Zeb D. Jonker, Gerard M. Ribbers, Ruud W. Selles, Jos N. van der Geest, Opher Donchin

**Affiliations:** 1 Department of Neuroscience, Erasmus MC, 3015 CN, Rotterdam, The Netherlands; 2 Department of Rehabilitation Medicine, Erasmus MC, 3015 CN, Rotterdam, The Netherlands; 3 Erasmus University College, 3011 HP, Rotterdam, The Netherlands; 4 Rijndam Rehabilitation Centre, 3015 LJ, Rotterdam, The Netherlands; 5 Department of Plastic and Reconstructive Surgery, Erasmus MC, 3015 CN, Rotterdam, The Netherlands; 6 Department of Biomedical Engineering and Zlotowski Center for Neuroscience, Ben Gurion University of the Negev, Be’er Sheva, 8499000 Israel

**Keywords:** Bayesian statistics, cerebellum, motor learning, noise, optimal control, visuomotor adaptation

## Abstract

Individual variations in motor adaptation rate were recently shown to correlate with movement variability or “motor noise” in a forcefield adaptation task. However, this finding could not be replicated in a meta-analysis of adaptation experiments. Possibly, this inconsistency stems from noise being composed of distinct components that relate to adaptation rate in different ways. Indeed, previous modeling and electrophysiological studies have suggested that motor noise can be factored into planning noise, originating from the brain, and execution noise, stemming from the periphery. Were the motor system optimally tuned to these noise sources, planning noise would correlate positively with adaptation rate, and execution noise would correlate negatively with adaptation rate, a phenomenon familiar in Kalman filters. To test this prediction, we performed a visuomotor adaptation experiment in 69 subjects. Using a novel Bayesian fitting procedure, we succeeded in applying the well-established state-space model of adaptation to individual data. We found that adaptation rate correlates positively with planning noise (*β* = 0.44; 95% HDI = [0.27 0.59]) and negatively with execution noise (*β* = –0.39; 95% HDI = [–0.50 –0.30]). In addition, the steady-state Kalman gain calculated from planning and execution noise correlated positively with adaptation rate (*r* = 0.54; 95% HDI = [0.38 0.66]). These results suggest that motor adaptation is tuned to approximate optimal learning, consistent with the “optimal control” framework that has been used to explain motor control. Since motor adaptation is thought to be a largely cerebellar process, the results further suggest the sensitivity of the cerebellum to both planning noise and execution noise.

## Significance Statement

Our study shows that the adaptation rate is optimally tuned to planning noise and execution noise across individuals. This suggests that motor adaptation is tuned to approximate optimal learning, consistent with “optimal control” approaches to understanding the motor system. In addition, our results imply sensitivity of the cerebellum to both planning noise and execution noise, an idea not previously considered. Finally, our Bayesian statistical approach represents a powerful, novel method for fitting the well-established state-space models that could have an influence on the methodology of the field.

## Introduction

As children we all learned: some of us move with effortless grace and others are frankly clumsy. Underlying these differences are natural variations in acquiring, calibrating, and executing motor skill, which have been related to genetic (Frank et al., 2009; Fritsch et al., 2010; McHughen et al., 2010) and structural (Tomassini et al., 2011) factors. Recently, it has been suggested that differences between individuals in the rate of motor adaptation (i.e. the component of motor learning responsible for calibrating acquired motor skills to changes in the body or environment; Shadmehr et al., 2010), correlate with movement variability, or motor noise (Wu et al., 2014). However, this finding was not supported by a recent meta-analysis of adaptation experiments (He et al., 2016). This inconsistency may arise because motor noise has multiple components with differing relations to adaptation rate. Our study characterizes the relationship between adaptation rate and motor noise and suggests that adaptation rate varies optimally between individuals in the face of multiple sources of motor variability.

**Figure 1.**
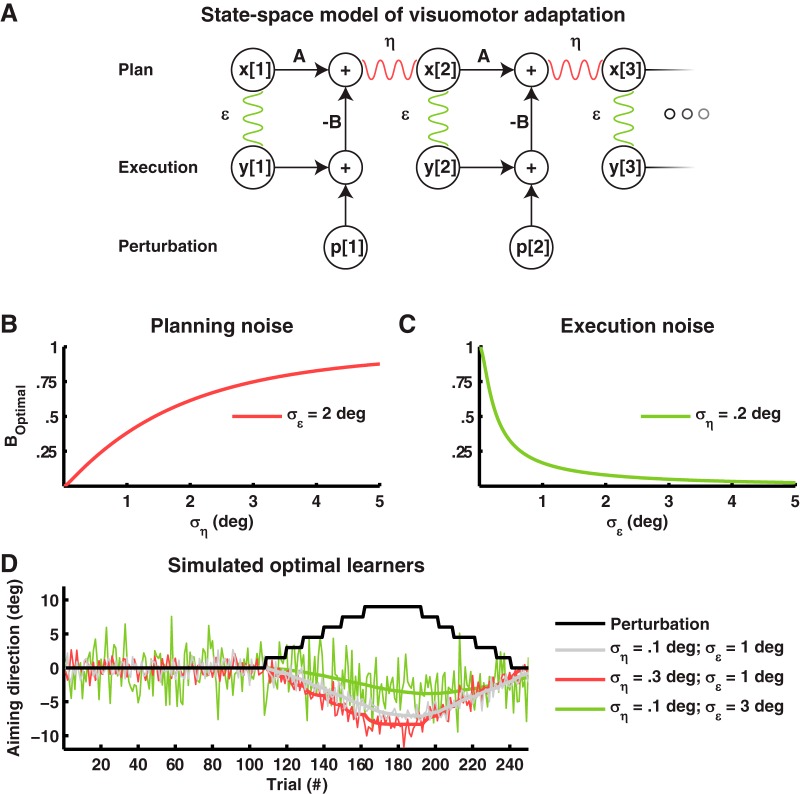
Planning and execution noise have opposing effects on visuomotor adaptation. ***A***, State-space model of visuomotor adaptation. The aiming angle on trial 2 x[2] is a linear combination of the aiming angle on the previous trial x[1] multiplied by a retentive factor A minus the error e[1] on the previous trial multiplied with adaptation rate B. In addition, the aiming angle is distorted by the random process η (planning noise). The actual movement angle y[2] is the aiming angle x[2] distorted by the random process ϵ (execution noise). The error e[1] is the sum of the movement direction y[1] and the external perturbation p[1]. ***B***, Planning noise and optimal adaptation rate $B_{Optimal}$ (defined as the Kalman gain). The optimal adaptation rate increases with planning noise $\sigma_{\eta }$. In this figure, $\sigma_{\epsilon }$ was kept constant at 2°. ***C***, Execution noise and optimal adaptation rate $B_{Optimal}$ (defined as the Kalman gain). The optimal adaptation rate decreases with execution noise $\sigma_{\epsilon }$. In this figure, $\sigma_{\eta }$ was kept constant at 0.2°. ***D***, Simulated optimal learners. At trial 110, a perturbation (black line) is introduced that requires the optimal learners to adapt their movement. The gray learner has low planning noise $\sigma_{\eta }=0.1{}^{\circ}$ and execution noise $\sigma_{\epsilon }=1{}^{\circ}$. The red learner has a higher planning noise $\sigma_{\eta }=0.3{}^{\circ}$ than the gray learner $\sigma_{\eta }=0.1{}^{\circ}$. This causes the red learner to adapt faster. The green learner has a higher execution noise than the gray learner $\sigma_{\epsilon }=3{}^{\circ}$. This causes the green learner to adapt more slowly. For all learners, the thick line shows the average, and the thin line, a single noisy realization.

Motor noise has many physiologic sources, such as motor preparation noise in (pre)motor networks, motor execution noise, and afferent sensory noise (Faisal et al., 2008). Modeling (Cheng and Sabes, 2006, 2007; van Beers, 2009) and physiologic (Churchland et al., 2006; Chaisanguanthum et al., 2014) studies have divided the multiple sources of motor noise into planning noise and execution noise (see Fig. 1*A*). Planning noise is believed to arise from variability in the neuronal processing of sensory information, as well as computations underlying adaptation and maintenance of the states in time (Cheng and Sabes, 2006, 2007). Indeed, electrophysiological studies in macaques show that activity in (pre)motor areas of the brain is correlated with behavioral movement variability (Churchland et al., 2006; Chaisanguanthum et al., 2014). Similar results have also been seen in humans using fMRI (Haar et al., 2017). In contrast, execution noise apparently originates in the sensorimotor pathway. In the motor pathway, noise stems from the recruitment of motor units (Harris and Wolpert, 1998; Jones et al., 2002; van Beers et al., 2004). Motor noise is believed to dominate complex reaching movements with reliable visual information (van Beers et al., 2004). In addition, sensory noise stems from the physical limits of the sensory organs and has been proposed to dictate comparably simpler smooth pursuit eye movements (Bialek, 1987; Osborne et al., 2005). Planning and execution noise might affect motor adaptation rate in different ways.

Motor adaptation has long been suspected to be sensitive to planning noise and execution noise. Models of visuomotor adaptation incorporating both planning and execution noise have been shown to provide a better account of learning than single noise models (Cheng and Sabes, 2006, 2007; van Beers, 2009). In addition, manipulating the sensory reliability by blurring the error feedback, effectively increasing the execution noise, can lower the adaptation rate (Baddeley et al., 2003; Burge et al., 2008; Wei and Körding, 2010; van Beers, 2012), whereas manipulating state estimation uncertainty by temporarily withholding error feedback, effectively increasing the planning noise, can elevate the adaptation rate (Wei and Körding, 2010). These studies not only suggest that adaptation rate is tuned to multiple sources of noise, but also indicate that this tuning process is optimal and can therefore be likened to a Kalman filter (Kalman, 1960). Possibly, differences in adaptation rate between individuals correlate with planning noise and execution noise according to the same principle, predicting faster adaptation for people with more planning noise and slower adaptation for people with more execution noise (He et al., 2016; Fig. 1*B*–*D*).

To test the relation between adaptation rate and planning noise and execution noise across individuals, we performed a visuomotor adaptation experiment in 69 healthy subjects. We fitted a state-space model of trial-to-trial behavior (Cheng and Sabes, 2006, 2007) using Bayesian statistics to extract planning noise, execution noise, and adaptation rate for each subject. We show that the adaptation rate is sensitive to both types of noise and that this sensitivity matches predictions based on Kalman filter theory.

## Materials and methods

### Subjects

We included 69 right-handed subjects between October 2016 and December 2016, without any medical conditions that might interfere with motor performance (14 men and 55 women; mean age = 21 years, range 18–35 years; mean handedness score = 79; range 45–100). Subjects were recruited from the Erasmus MC University Medical Center and received a small financial compensation. The study was performed in accordance with the Declaration of Helsinki and approved by the medical ethics committee of the Erasmus MC University Medical Center.

### Experimental procedure

**Figure 2.**
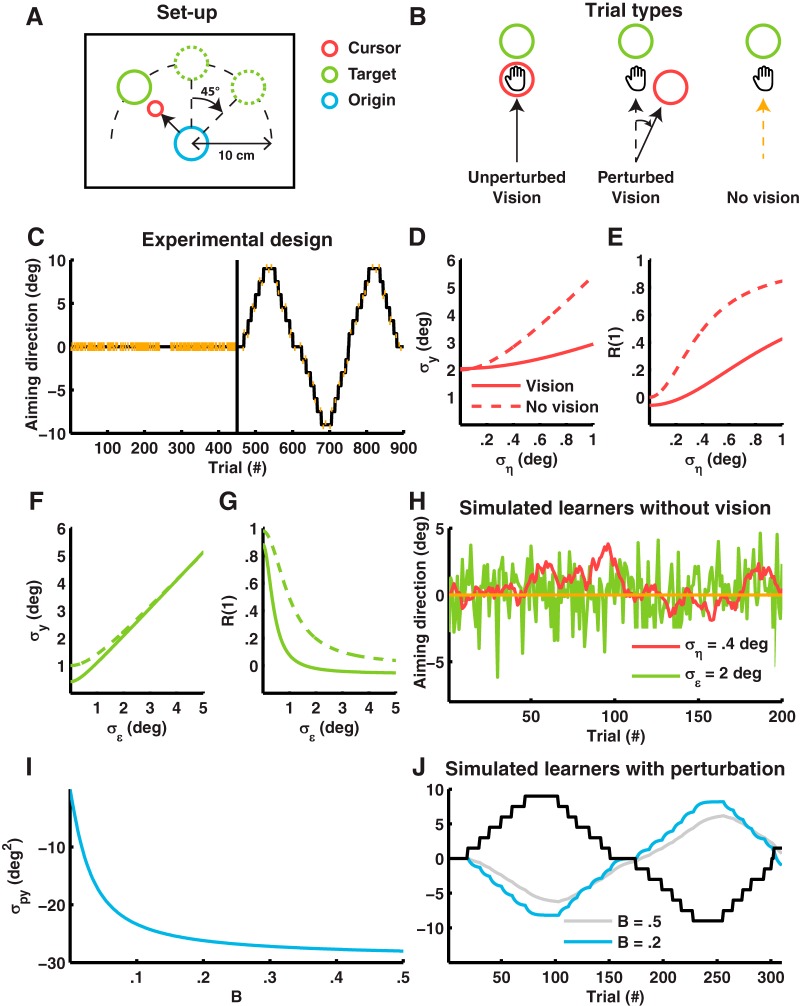
Measurements of planning and execution noise and adaptation rate in a visuomotor adaptation experiment. ***A***, Setup. The projection screen displayed the location of the robotic handle (“the cursor”), start location of the movement (“the origin”), and target of the movement (“the target”) on a black background. The position of the origin on the screen was fixed throughout the experiment, while the target was placed 10 cm from the origin at an angle of –45°, 0°, or 45°. ***B***, Trial types. The experiment included vision unperturbed and perturbed trials and no-vision trials. In vision unperturbed trials, the cursor was shown at the position of the handle during the movement. The cursor was also visible in vision perturbed trials, but at a predefined angle from the vector connecting the origin and the handle. In no-vision trials, the cursor was turned off when movement onset was detected and therefore only visible at the start of movement to help subjects keep the cursor at the origin. ***C***, Experimental design. The baseline block consisted of 225 vision unperturbed trials and 225 no-vision trials (indicated by vertical red lines). The perturbation block had 50 no-vision trials and 400 vision trials, with every block of nine trials containing 1 no-vision trial. Most vision trials were perturbed vision trials whose perturbation magnitudes formed a staircase running from –9° to 9°. ***D***, Simulation of planning noise $\sigma_{\eta }$ and standard deviation $\sigma_{y}$ of the movement angle. $\sigma_{y}$ increases with $\sigma_{\eta }$. Calculated for A=0.98 and $\sigma_{\epsilon }=2{}^{\circ}$ with B=0.2 for the solid line and B=0 for the dashed line. ***E***, Simulation of planning noise $\sigma_{\eta }$ and lag-1 autocorrelation R(1) of the movement angle. R(1) increases with $\sigma_{\eta }$. Calculated for A=0.98 and $\sigma_{\epsilon }=2{}^{\circ}$ with B=0.2 for the solid line and B=0 for the dashed line. ***F***, Simulation of execution noise $\sigma_{\epsilon }$ and standard deviation $\sigma_{y}$ of the movement angle. $\sigma_{y}$ increases with $\sigma_{\epsilon }$. Calculated for A=0.98 and $\sigma_{\eta }=0.2{}^{\circ}$ with B=0.2 for the solid line and B=0 for the dashed line. ***G***, Simulation of execution noise $\sigma_{\epsilon }$ and lag-1 autocorrelation R(1) of the movement angle. R(1) decreases with $\sigma_{\epsilon }$. Calculated for A=0.98 and $\sigma_{\eta }=0.2{}^{\circ}$ with B=0.2 for the solid line and B=0 for the dashed line. **H.** Simulated learners without vision. The green and red traces show a single realization of two learners with either high planning noise (red learner $\sigma_{\eta }=0.4{}^{\circ}$ and $\sigma_{\epsilon }=0{}^{\circ}$) or high execution noise (green learner $\sigma_{\eta }=0{}^{\circ}$ and $\sigma_{\epsilon }=2{}^{\circ}$). Both sources increase the movement noise, but planning noise leads to correlated noise, whereas execution noise leads to uncorrelated noise. This property can be seen from the relation between sequential trials. For the red learner, sequential trials are often in the same (positive or negative) direction. For the green learner, sequential trials are in random directions. This is captured by the lag-1 autocorrelation. ***I***, Simulation of $\sigma_{py}$ between the perturbation p and movement angle y, and adaptation rate B. $\sigma_{py}$ gets more negative for increasing B (simulated with A=0.98). ***J***, Simulated learners with perturbation. The gray and blue lines show a simulated slow (A=0.98, B=0.05) and fast (A=0.98, B=0.2) learner. The fast learner tracks the perturbation signal more closely than the slow learner. This property is captured by the covariance between the perturbation and the movement angle.

Subjects were seated in front of a horizontal projection screen while holding a robotic handle in their dominant right hand (Donchin et al., 2012). The projection screen displayed the location of the robotic handle (“the cursor”; yellow circle 5-mm radius), start location of the movement (“the origin”, white circle 5-mm radius), and target location of the movement (“the target”, white circle 5-mm radius) on a black background (see Fig. 2*A*). The position of the origin on the screen was fixed throughout the experiment, ∼40 cm in front of the subject at elbow height, while the target was placed 10 cm from the origin at an angle of –45°, 0°, or 45°. To remove direct visual feedback of hand position, subjects wore an apron that was attached to the projection screen around their necks.

Subjects were instructed to make straight shooting movements from the origin toward the target and to decelerate only when they passed the target. A trial started with the presentation of the target and ended when the distance between the origin and cursor was at least 10 cm or when trial duration exceeded 2 s. At this point, movements were damped with a force cushion (damper constant 3.6 Ns/m, ramped up over 7.5 ms) and the cursor was displayed at its last position until the start of the next trial to provide position error feedback. Furthermore, timing feedback was given to keep trial duration (see definition below) in a tight range. The target dot turned blue if trial duration on a particular trial was too long (>600 ms) and red if trial duration was too short (<400 ms) and remained white if trial duration was in the correct time range (400–600 ms). During presentation of position and velocity feedback, the robot pushed the handle back to the starting position. Forces were turned off when the handle was within 0.5 cm from the origin. Concurrently, the cursor was projected at the position of the handle again and subjects had to keep the cursor within 0.5 cm from the origin for 1 s to start the next trial.

The experiment included vision unperturbed, vision perturbed, and no-vision trials (see Fig. 2*B*). In vision unperturbed trials, the cursor was shown at the position of the handle during the movement. The cursor was also visible in vision perturbed trials but at a predefined angle from the vector connecting the origin and the handle. In no-vision trials, the cursor was turned off when movement onset was detected (see below) and was visible only at the start of the trial to help subjects keep the cursor at the origin.

The entire experiment lasted 900 trials with all three target directions (angle of –45°, 0°, or 45°) occurring 300 times in random order. The three different trial types were used to build a baseline and a perturbation block (see Fig. 2*C*). We designed the baseline block to obtain (1) reliable estimates of the noise parameters and (2) variance statistics (standard deviation and lag-1 autocorrelation of the movement angle) related to the noise parameters. Therefore, we included a large number of no-vision trials (225 no-vision trials) as well as vision unperturbed trials (225 vision unperturbed trials). The order of the vision unperturbed trials and no-vision trials was randomized except for trials 181–210 (no-vision trials) and trials 241–270 (vision unperturbed trials). We designed the perturbation block to obtain (1) reliable estimates of the adaptation parameters and (2) variance statistics related to trial-to-trial adaptation (covariance between perturbation and movement angle). The perturbation block consisted of a large number of vision trials (400 vision trials) and a small number of no-vision trials (50 no-vision trials), with every block of nine trials containing one no-vision trial. Every 8 to 12 trials, the perturbation angle changed with an incremental 1.5° step. These steps started in the positive direction until reaching 9° and then switched sign to continue in the opposite direction until reaching –9°. This way, a perturbation signal was constructed with three “staircases” lasting 150 trials each (see Fig. 2*C*). Design of the gradual perturbation was optimized to provide a “rich” input for system identification, without sacrificing the consistency of the signal too much, as this has been shown to negatively affect the adaptation rate (Gonzalez Castro et al., 2014; Herzfeld et al., 2014), and is similar to the perturbation used by Cheng and Sabes (2007). The experiment was briefly paused every 150 trials.

### Data collection

The experiment was controlled by a C++ program developed in-house. Position and velocity of the robot handle were recorded continuously at a rate of 500 Hz. Velocity data were smoothed with an exponential moving average filter (smoothing factor = 0.18 s). Trials were analyzed from movement start (defined as the time point when movement velocity exceeds 0.03 m/s) to movement end (defined as the time point when the distance from the origin is equal to or larger than 9.5 cm). Reaction time was defined as the time from trial start until movement start, movement duration as the time from movement start until trial end and trial duration as the time from trial start until trial end. Movement angle was calculated as the signed (+ or –) angle in degrees between the vector connecting origin and target and the vector connecting robot handle position at movement start and movement end. The clockwise direction was defined as positive. Peak velocity was found by taking the maximum velocity in the trial interval. Trials with (1) a maximal displacement below 9.5 cm, (2) an absolute movement direction larger than 30°, or (3) a duration longer than 1 s were removed from further analysis (2% of data).

### Visuomotor adaptation model

Movement angle was modeled with the following state-space equation (see Fig. 1*A*; Cheng and Sabes, 2006, 2007):

(1) $$x\left[n+1\right]=Ax\left[n\right]-Be[n]+\eta$$

(2) $$y\left[n\right]=x\left[n\right]+\epsilon$$

(3) $$e\left[n\right]=y\left[n\right]+p[n]$$

(4) $$\eta ∼N\left(0,\sigma_{\eta }^{2}\right),\epsilon ∼N(0,\sigma_{\epsilon }^{2})$$

In this model, x[n] is the aiming angle (the movement plan), and y[n] is the movement angle (the actually executed movement). Error *e* [n] on a particular trial is the sum of y[n] and the perturbation p[n]. The learning terms are A, which represents retention of the aiming angle over trials, and adaptation rate B, the fractional change from error e[n]. The movement angle is affected by planning noise process η, modeled as a zero-mean Gaussian with standard deviation $\sigma_{\eta }$, and execution noise process ϵ, modeled as a zero-mean Gaussian with standard deviation $\sigma_{\epsilon }$.

### Statistics

Our statistical approach is a Bayesian approach (an excellent introduction to Bayesian statistics for a nontechnical audience can be found in Kruschke (2010)). We used this approach to fit the state-space model described in Eqs. (1)–(4) because it offers a number of advantages over the expectation-maximization algorithm used in previous studies (Cheng and Sabes, 2006, 2007). Perhaps the most important advantage of the Bayesian approach is that it naturally allows hierarchical modeling that shares data across subjects, allowing greater regularization of the parameter fits for each subject, as well as simultaneous estimates of the population distribution of the parameters (Browne and Draper, 2006; Gelman, 2006). In a classic approach, each subject’s parameters are generally estimated independently, and the uncertainty in those estimates is often not propagated forward when calculating population estimates. Indeed, the output of a Bayesian approach is not the best possible estimate of the parameter or even a maximum-likelihood estimate with a confidence interval, but rather a sampling from the parameter’s probability distribution given the data (Kruschke and Liddell, 2018a). This allows the analysis to naturally refocus on parameter uncertainty rather than focusing on point estimates (Kruschke, 2013; Wagenmakers et al., 2014; Kruschke and Liddell, 2018b). The difficulty with point estimates has been a focus of much debate in the current discussion of the reproducibility crisis in science (Ioannidis, 2005; Cumming, 2014). The Bayesian approach also estimates the hidden (state) variables simultaneously with the parameters, rather than creating a somewhat arbitrary distinction between imputation and estimation (Carlin et al., 1992; Carter and Kohn, 1994). This allows analysis of how the state variable estimates change with the parameter estimates, an analysis that is tricky to do with an expectation-maximization approach. Finally, the Bayesian approach allows great flexibility in specifying the form of the model (Kruschke and Liddell, 2018a). This can be useful in defining constraints on the model parameters or transforming variables to lie in more relevant parameter spaces, as defined below.

Modern Bayesian approaches rely on a family of algorithms called the Markov chain Monte Carlo (MCMC) algorithms (Andrieu et al., 2003). These algorithms require definitions of the likelihood function (how the data would be generated if we knew the parameters), the prior probability for the parameters (generally chosen to be broad and uninformative, but see below), and return samples from the posterior joint-probability function of the parameters. Thus, once the model and priors are specified, the output of the MCMC algorithm is a large matrix where each row is a sample and each column is one of the parameters in the model. These samples can be, then, summarized in different ways to generate parameter estimates (usually the mean of the samples but often the mode) and regions of uncertainty (very often a 95% region called the high-density interval (HDI) which contains 95% of the posterior samples but also obeys the criterion that every sample in the HDI is more probable than every sample outside of it). They can also be used to assess asymmetry in the parameter distributions and covariance in the parameter estimates.

As outlined above, the Bayesian approach to state-space modeling we have taken requires us to define priors on the model parameters. We will justify our choices in the following section. The adaptation parameters B[s] and retention parameters A[s] were sampled in the logistic space instead of the regular 0-1 space:

(5) $$A[s]∼\frac{1}{1+\exp\left(-N\left(\mu_{A},\sigma_{A}^{2}\right)\right)},B\left[s\right]∼\frac{1}{1+\exp\left(-N\left(\mu_{B},\sigma_{B}^{2}\right)\right)}$$

The logistic space spreads the range from 0-1 all the way from -∞ to +∞. This means that the distance between 0.1 and 0.01 and 0.001 are all similar in the logistic space, as are the distances between 0.9, 0.99 and 0.999. This space, thus, reflects much more accurately the real effects of changes in the parameter than if we sampled in the untransformed space. This leads to much better sampling behavior and, thus, greater accuracy and less bias in the results. The priors for A[s] and B[s] were not actually specified in the description of the model. Only their shape was determined (normal in the logistic space). The actual prior was chosen by sampling hyperparameters for these normal distributions. For the hyperparameters, we did need to choose a specific prior, and here we choose highly uninformative priors to allow the posterior distribution to be influenced primarily by the data:

(6) $$\mu_{A}∼N\left(0,10^{3}\right),\mu_{B}∼N(0,10^{3})$$

(7) $$\sigma_{A}^{2}∼\sigma_{B}^{2}∼1/\Gamma (10^{-3},10^{-3})$$

The sensitivity analysis (described below) showed that the choice to sample A[s] and B[s] from a normal distribution in the logistic space had no strong effect on the results. Following the standard Bayesian approach (Kruschke, 2010), we sampled the precision (inverse of the variance) and used a very broad gamma distribution as a prior for the precision:

(8) $$\sigma_{\eta }^{2}\left[s\right]∼1/\Gamma \left(10^{-3},10^{-3}\right),\sigma_{\epsilon }^{2}\left[s\right]∼1/\Gamma \left(10^{-3},10^{-3}\right)$$

One reason the gamma distribution is a popular prior for the precision is that it is a conjugate prior which makes the algorithm more efficient. In any case, other choices of prior did not change our results in a meaningful way (see sensitivity analysis below).

MCMC sampling for the Bayesian state-space model was implemented in OpenBUGS (v. 3.2.3, OpenBUGS Foundation, available from: http://www.openbugs.net/w/Downloads) with three 50,000 samples chains and 20,000 burn-in samples. A single estimate per subject s was made for A[s] and B[s], $\sigma_{\eta }^{2}[s]$ and $\sigma_{\epsilon }^{2}[s]$. We used all 150,000 MCMC samples that represent the posterior distribution of the model parameters B[s], $\sigma_{\eta }[s]$, and $\sigma_{\epsilon }[s]$ given the data to calculate linear regressions and correlations between the model parameters across subjects. Results were presented as the mode of the effect size (either the correlation coefficient *r* or regression coefficient *β*) with 95% HDIs. Parameter estimates are plotted as the mode with 68% HDIs, similar to the standard deviation interval.

To demonstrate the test-retest properties of the Bayesian state-space model, we simulated two datasets with 50 learners on the visuomotor adaptation task outlined above. The first (optimal) dataset was simulated by drawing model parameters from the following distributions: $A[s]∼N\left(0.97,10^{-4}\right)$, $\sigma_{\eta }[s]∼N\left(0.6,0.04\right)$, and $\sigma_{\epsilon }[s]∼N\left(3,0.5625\right)$, and calculating B[s] as the Kalman gain. The goal of this analysis was to determine the test-retest correlations of the model parameters B[s], $\sigma_{\eta }[s]$, and $\sigma_{\epsilon }[s]$ and the ability to correctly estimate the relations between B[s] and the noise parameters. For the second (permuted) dataset, A[s], $\sigma_{\eta }[s]$, and $\sigma_{\epsilon }[s]$ were kept constant but B[s] was permuted between learners. The motivation for this analysis was to show that our Bayesian state-space model does not introduce false relations between B and the noise parameters.

To evaluate the sensitivity of the main results to alternate prior distributions for the Bayesian state-space model, we repeated the entire analysis with (alternative priors 1) *t*-distributions with the hyperparameter for the degrees of freedom sampled from an exponential distribution (in line with recommendations from Kruschke (2013)) as priors for A[s] and B[s]; (alternative priors 2) *t*-distributions as priors for A[s] and B[s], and uniform distributions in the range [0,20] as priors for $\sigma_{\eta }$ and $\sigma_{\epsilon }$ (in line with recommendations from Gelman (2006)); and (alternative priors 3) beta distributions with hyperparameters sampled from gamma distributions as priors for A[s] and B[s] and uniform distributions as priors for $\sigma_{\eta }$ and $\sigma_{\epsilon }$. Finally, we addressed the concern that the between-subjects correlations of the model parameters might arise from within-subject correlations of the model parameters by permuting the MCMC samples differently for each parameter and recalculating the correlation and regression coefficients. The permuted distribution of the model parameters has the property that all correlations between the parameters within subjects are zero.

### Code accessibility

BUGS/JAGS code for the Bayesian state-space model can be accessed without restrictions at: https://github.com/rickvandervliet/Bayesian-state-space.

## Results

### Simulations

We designed a visuomotor adaptation task (Tseng et al., 2007) to (1) fit the state-space model of adaptation and (2) investigate the validity of the parameter estimates B[s], $\sigma_{\eta }[s]$, and $\sigma_{\epsilon }[s]$ by correlating the estimates with the variance statistics of the data (see Fig. 2*A–C*).

The baseline block was designed to extract the standard deviation and the lag-1 autocorrelation of the movement direction and relate these measures to the parameter estimates of $\sigma_{\eta }[s]$ and $\sigma_{\epsilon }[s]$. The standard deviation and lag-1 autocorrelation in our baseline block are well approximated by the following expressions:

(9) $$\sigma_{y}=\sqrt{\left(\sigma_{\epsilon }^{2}+\sum_{k=0}^{\infty }{\left(A-B\right)}^{2k}\sigma_{\eta }^{2}+\sum_{k=0}^{\infty }{\left(A-B\right)}^{2k}B^{2}\sigma_{\epsilon }^{2}\right)}$$

(10) $$R(1)=\frac{\sum_{k=0}^{\infty }{\left(A-B\right)}^{2k+1}\sigma_{\eta }^{2}+B\sigma_{\epsilon }^{2}+\sum_{k=0}^{\infty }{\left(A-B\right)}^{2k+1}B^{2}\sigma_{\epsilon }^{2}}{\sum_{k=0}^{\infty }{A^{k}(A-B)}^{k}\sigma_{\eta }^{2}+\sigma_{\epsilon }^{2}+\sum_{k=0}^{\infty }{A^{k}(A-B)}^{k}B^{2}\sigma_{\epsilon }^{2}}$$

In addition, we included a control segment of 30 trials without vision (B = 0), to calculate estimates of the standard deviation and lag-1 autocorrelation which are independent of the adaptation rate B:

(11) $$\sigma_{y}=\sqrt{\left(\sigma_{\epsilon }^{2}+\sum_{k=0}^{\infty }A^{2k}\sigma_{\eta }^{2}\right)}$$

(12) $$R(1)=\frac{\sum_{k=0}^{\infty }{(A}^{2k+1}\sigma_{\eta }^{2})}{\sigma_{\epsilon }^{2}+\sum_{k=0}^{\infty }A^{2k}\sigma_{\eta }^{2}}$$

For both the expressions with vision (9)–(10) (solid lines) and without vision (11)–(12) (dashed lines), standard deviation $\sigma_{y}$ increases with planning noise $\sigma_{\eta }$ (see simulations in Fig. 2*D*) and execution noise $\sigma_{\epsilon }$ (see simulations in Fig. 2*F*) whereas lag-1 autocorrelation $R\left(1\right)$ increases with planning noise $\sigma_{\eta }$ (see simulations in Fig. 2*E*) but decreases with execution noise $\sigma_{\epsilon }$ (see simulations in Fig. 2*G*), with the strongest correlations between $\sigma_{y}$ and $\sigma_{\epsilon }$, and $R\left(1\right)$ and $\sigma_{\eta }$. We therefore expected similar relations between the noise parameters $\sigma_{\eta }[s]$ and $\sigma_{\epsilon }[s]$, and the standard deviation $\sigma_{y,baseline}[s]$ and lag-1 autocorrelation $R_{Baseline}\left(1\right)[s]$ of the baseline block (see simulations of planning and execution noise in the baseline block in Fig. 2*H*).

The perturbation block was designed to extract the covariance $\sigma_{py}$ between the perturbation and the movement angle from the data and relate this parameter to the adaptation rate B. The covariance $\sigma_{py}$ depends solely on the learning parameters A and B and becomes increasingly negative for higher adaptation rates because learning is compensatory (see simulations in Fig. 2*I*). Therefore, we expected a similar relation between the covariance $\sigma_{py}[s]$ and adaptation rate B[s] in the perturbation block of our experiment (see simulations of two learners with a low or high adaptation rate in Fig. 2*J*).

**Figure 3.**
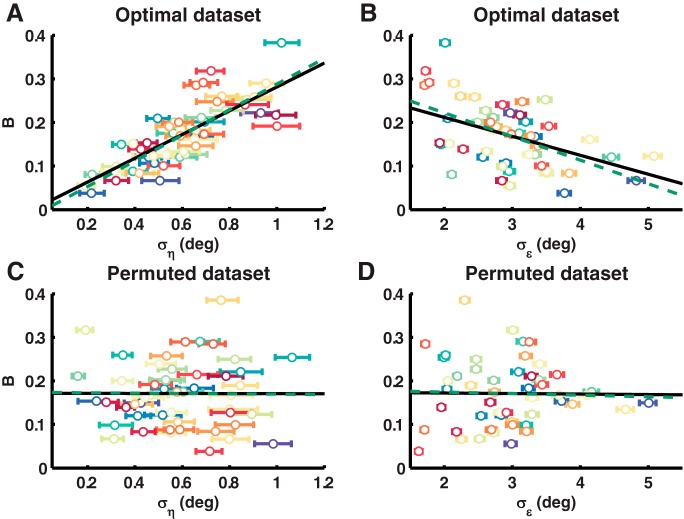
Test-retest properties of the Bayesian state-space model. ***A***, ***B***, Regression of $B\left[s\right]$ onto $\sigma_{\eta }\left[s\right]$ (***A***) and $\sigma_{\epsilon }\left[s\right]$ (***B***) for the simulated optimal dataset. ***C***, ***D***, Regression of $B\left[s\right]$ onto $\sigma_{\eta }\left[s\right]$ (***C***) and $\sigma_{\epsilon }\left[s\right]$ (***D***) for the simulated permuted dataset. Parameter estimates with 68% HDIs are shown for every simulated learner as a dot with error bars. The black solid line shows the regression on the model parameters estimated with the Bayesian state-space model, the green dashed line the regression on the original model parameters.

Next, we designed a Bayesian state-space model to estimate the model parameters. To demonstrate the test-retest properties of this approach, we simulated one dataset with optimal learners and one dataset wherein the adaptation rate of the optimal dataset was permuted across learners. Excellent test-retest correlations were found in both the optimal dataset (B[s] *r* = 1.00; 95% HDI = [1.00 1.00], $\sigma_{\eta }[s]$ *r* = 0.89; 95% HDI = [0.85 0.93], and $\sigma_{\epsilon }[s]$ *r* = 0.99; 95% HDI = [0.98 0.99]) and the permuted dataset (B[s] *r* = 1.00; 95% HDI = [1.00 1.00], $\sigma_{\eta }[s]$ *r* = 0.90; 95% HDI = [0.86 0.93], and $\sigma_{\epsilon }[s]$ *r* = 0.99; 95% HDI = [0.98 0.99]). In the optimal dataset, the Bayesian state-space model was able to uncover the relations between B[s] and the noise parameters $\sigma_{\eta }[s]$ *β* = 0.73; 95% HDI = [0.68 0.77] (see Fig. 3*A*) and $\sigma_{\epsilon }[s]$ *β* = –0.44; 95% HDI = [–0.51 –0.38]), which were 0.81 and –0.53 in the simulated data (see Fig. 3*B*). In the permuted dataset, the Bayesian state-space model did not falsely introduce relations between B[s] and the noise parameters $\sigma_{\eta }[s]$ *β* = 0; 95% HDI = [–0.09 0.08] (see Fig. 3*C*) and $\sigma_{\epsilon }[s]$ *β* = –0.01; 95% HDI = [–0.04 0.02]), as they were –0.01 and –0.04 in the original dataset (see Fig. 3*D*). Therefore, the Bayesian state-space model can reliably estimate the model parameters and the regression coefficients between the noise terms and the adaptation rate.

### Experimental results

**Figure 4.**
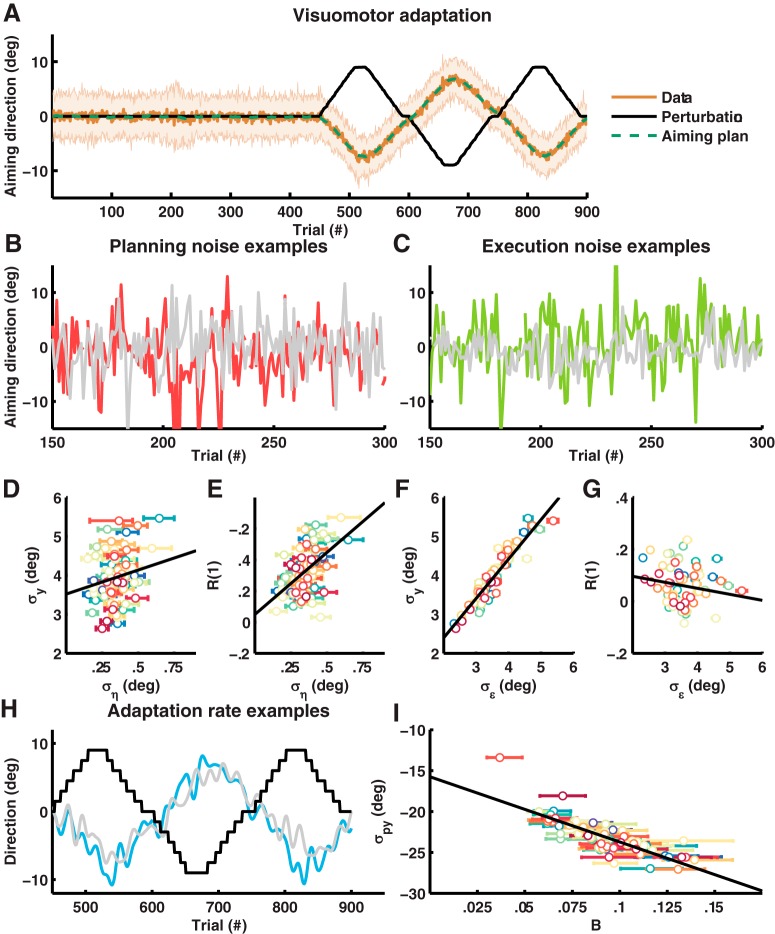
State-space model of visuomotor adaptation. ***A***, Visuomotor adaptation. Average movement angle of the 69 subjects with standard deviations are shown in brown tone colors. The black line indicates the average perturbation signal, and the green line, the average posterior estimate of the aiming angle. ***B***, Planning noise examples. The gray line shows a subject with low planning noise ($\sigma_{\eta }=0.15{}^{\circ}\;\sigma_{\epsilon }=4.6{}^{\circ}$), and the red line, a subject with high planning noise ($\sigma_{\eta }=0.65\,{}^{\circ}\;\sigma_{\epsilon }=4.6{}^{\circ}$). ***C***, Execution noise examples. The gray line shows a subject with low execution noise ($\sigma_{\eta }=0.36\,{}^{\circ}\;\sigma_{\epsilon }=2.3{}^{\circ}$), and the green line, a subject with high execution noise ($\sigma_{\eta }=0.29\,{}^{\circ}\;\sigma_{\epsilon }=5.0{}^{\circ}$). ***D***, Relation between the parameter estimate $\sigma_{\eta }$ and baseline measure $\sigma_{y,baseline}$. The black line is a linear regression of $\sigma_{y,baseline}[s]$ onto $\sigma_{\eta }[s]$ and $\sigma_{\epsilon }[s]$ for average $\sigma_{\epsilon }[s]$. ***E***, Relation between the parameter estimate $\sigma_{\eta }$ and baseline measure $R{\left(1\right)}_{baseline}$. The black line is a linear regression of $R{\left(1\right)}_{baseline}[s]$ onto $\sigma_{\eta }[s]$ and $\sigma_{\epsilon }[s]$ for average $\sigma_{\epsilon }[s]$. ***F***, Relation between the parameter estimate $\sigma_{\epsilon }$ and baseline measure $\sigma_{y,baseline}$. The black line is a linear regression of $\sigma_{y,baseline}[s]$ onto $\sigma_{\eta }[s]$ and $\sigma_{\epsilon }[s]$ for average $\sigma_{\eta }[s]$. ***G***, Relation between the parameter estimate $\sigma_{\epsilon }$ and baseline measure $R{\left(1\right)}_{baseline}$. The black line is a linear regression of $R{\left(1\right)}_{baseline}[s]$ onto $\sigma_{\eta }[s]$ and $\sigma_{\epsilon }[s]$ for average $\sigma_{\eta }[s]$. ***H***, Adaptation rate examples. The thick lines show a slow (gray, B=0.055) and fast (blue, B=0.14) subject smoothed with a 6th-order Butterworth filter. The black shows the perturbation signal for the fast subject. ***I***, Relation between the parameter estimate B[s] and perturbation block estimate $\sigma_{py}[s]$. Parameter estimates and 68% HDIs are shown for every subject as a dot with error bars.

Sixty-nine subjects performed the visuomotor adaptation task outlined above. Overall, participants started moving 230 ms, IQR = [211 254] ms, after target presentation and completed the movement in 290 ms, IQR = [251 320] ms, resulting in a trial duration of 520 ms, IQR = [500 534] ms with 87% of trials IQR = [84 95]% in the correct time window between 400 and 600 ms. Standard deviation of movement angle calculated across the 69 subjects illustrates the differences in movement behavior between people (Fig. 4*A*). The group average aiming angle $x\left[n\right]$, calculated from 1,000 samples of the posterior distribution using the model (green dotted line), shows good agreement with the group average movement angle calculated directly from the data (brown solid line).

Fig. 4*B*, *C* show example subjects with low or high planning noise $\sigma_{\eta }[s]$ (see Fig. 4*B*) and low or high execution noise $\sigma_{\epsilon }[s]$ (see Fig. 4*C*). We calculated the standard deviation and lag-1 autocorrelation using all trials in the baseline block and regressed these estimates onto $\sigma_{\eta }[s]$ and $\sigma_{\epsilon }\left[s\right]$. Agreeing with our group-level predictions (see Fig. 2*D–G*), we found a positive relation between planning noise $\sigma_{\eta }[s]$ and standard deviation $\sigma_{y,baseline}[s]$ (*β* = 0.18; 95% HDI = [0.11 0.24]; see Fig. 4*D*), between planning noise $\sigma_{\eta }[s]$ and lag-1 autocorrelation $R_{Baseline}\left(1\right)[s]$ (*β* = 0.42; 95% HDI = [0.29 0.55]; see Fig. 4*E*) and between execution noise $\sigma_{\epsilon }$[s] and standard deviation $\sigma_{y,baseline}[s]$ (*β* = 0.91; 95% HDI = [0.87 0.94]; see Fig. 4*F*) and a negative relation between execution noise $\sigma_{\epsilon }[s]$ and lag-1 autocorrelation $R_{Baseline}\left(1\right)[s]$ (*β* = –0.14; 95% HDI = [–0.24 –0.07]; see Fig. 4*G*). Next, we calculated the standard deviation and lag-1 autocorrelation of trials 181–210 only, which are no-vision trials where adaptation rate B = 0. Here, we found similar correlations between (1) planning noise $\sigma_{\eta }[s]$ and standard deviation$\sigma_{y,novision}[s]$ (*β* = 0.12; 95% HDI = [–0.04 0.27]; (2) planning noise $\sigma_{\eta }[s]$ and lag-1 autocorrelation $R_{Novision}\left(1\right)[s]$ (*β* = 0.22; 95% HDI = [0.07 0.35]; (3) execution noise $\sigma_{\epsilon }$[s] and standard deviation $\sigma_{y,novision}[s]$ (*β* = 0.44; 95% HDI = [0.39 0.49]), and (4) execution noise $\sigma_{\epsilon }[s]$ and lag-1 autocorrelation $R_{Novision}\left(1\right)[s]$ (*β* = –0.04; 95% HDI = [–0.10 –0.01]). Example subjects with a low and high adaptation rate are shown in Fig. 4*H*. Again, according to the model prediction (see Fig. 2*I*), we found a negative relation between adaptation rate B[s] and covariance $\sigma_{py}[s]$ on a group level (*r* = –0.69; 95% HDI = [–0.78 –0.60]; see Fig. 4*I*).

**Figure 5.**
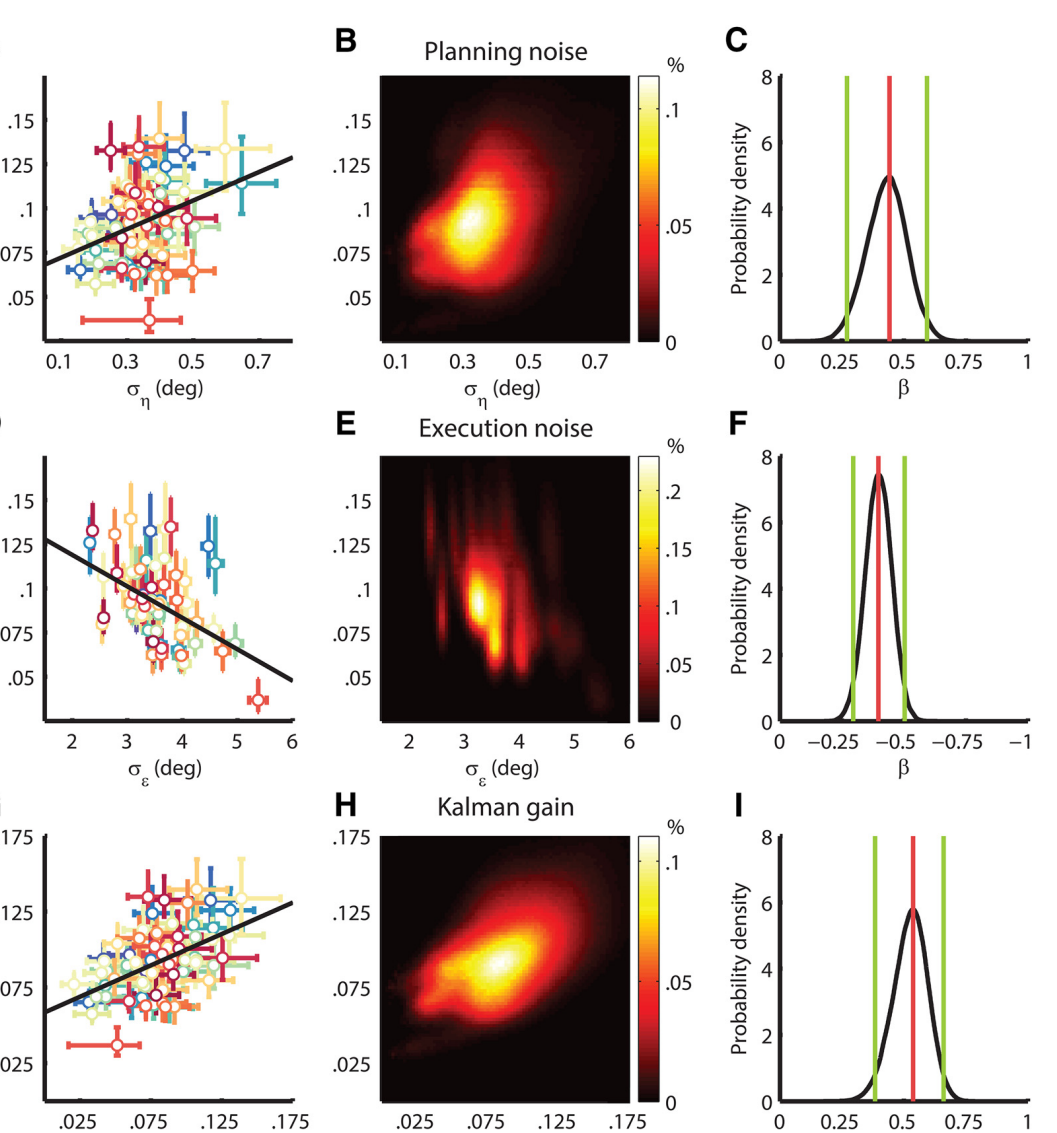
Relation between noise and adaptation rate. ***A***, ***D***, ***G***, Scatter plots of individual parameter estimates. Parameter estimates and 68% HDIs are shown for every subject as a dot with error bars. The black line is a linear regression of B[s] onto $\sigma_{\eta }[s]$ and $\sigma_{\epsilon }[s]$ for average $\sigma_{\epsilon }[s](A)$, a linear regression of B[s] onto $\sigma_{\eta }[s]$ and $\sigma_{\epsilon }[s]$ for average $\sigma_{\eta }[s]$ (***D***) and the correlation between K[s] and B[s] (***G***). ***B***, ***E***, ***H***, Heatmaps of the parameter estimate distributions. The heatmaps illustrate the distribution of the parameter estimates for the entire population of 69 subjects. The intensity represents the percentage of samples in a specific range for (**B**) $\sigma_{\eta }[s]$ and B[s] (***B***), $\sigma_{\epsilon }[s]$ and B[s] (***E***), and K[s] and B[s] (***H***). ***C***, ***F***, ***I***, Effect size densities. The black line represents the probability density of the regression coefficient for B[s] and $\sigma_{\eta }[s]$ (***C***), the regression coefficient for B[s] and $\sigma_{\epsilon }[s]$ (***F***), and the correlation coefficient for B[s] and K[s] (***I***). The green lines indicate the 95% HDIs. The red line shows the mode.

Next, we investigated the relation between adaptation rate and the noise terms. The results are illustrated with scatterplots of the parameter estimates for individual subjects (Fig. 5, left column), heatmaps of the parameter estimate distributions for the entire population (Fig. 5, middle column), and line plots of the regression and correlation coefficient densities (Fig. 5, right column). We regressed $B\left[s\right]$ onto $\sigma_{\eta }[s]$ and $\sigma_{\epsilon }\left[s\right]$ and found a positive relation between $\sigma_{\eta }[s]$ and $B\left[s\right]$ (*β* = 0.44; 95% HDI = [0.27 0.59]; see Fig. 5*A–C*) and a negative relation between $\sigma_{\epsilon }[s]$ and $B\left[s\right]$ (*β* = –0.39; 95% HDI = [–0.50 –0.30]; see Fig. 5*D–F*) with a variance explained of 0.32; 95% HDI = [0.19 0.45]. This finding indicates that a significant proportion of the difference in adaptation rate between individuals can be explained from differences in their planning and execution noise with the direction of the correlations in agreement with Kalman filter theory (see Fig. 1*B*, *C*). In addition, we determined the steady-state Kalman gain for every subject from $A\left[s\right]$, $\sigma_{\eta }[s]$, and $\sigma_{\epsilon }\left[s\right]$ and correlated the steady-state Kalman gain with B[s]. Steady-state Kalman gain was calculated by solving the Riccati equation for the steady-state covariance $P_{\infty }[s]$:

(13) $${A[s]}^{T}P_{\infty }[s]A[s]-P_{\infty }[s]-{A\left[s\right]}^{T}P_{\infty }[s]{\left(P_{\infty }\left[s\right]+\sigma_{\epsilon }{\left[s\right]}^{2}\right)}^{-1}P_{\infty }[s]A[s]+\sigma_{\eta }{\left[s\right]}^{2}=0$$

(14) $$K[s]=P_{\infty }[s](P_{\infty }[s]+\sigma_{\epsilon }{\left[s\right]}^{2}{)}^{-1}$$

On a group level, the Kalman gain was a good approximation for the adaptation rate as the difference between the mean K[s], and the mean $B\left[s\right]$ normalized with respect to the mean $B\left[s\right]$ was 10%; 95% HDI = [6.6 14]%. On an individual level, we found a positive correlation between steady-state Kalman gain K[s] and B[s] (*r* = 0.54; 95% HDI = [0.38 0.66]; see Fig. 5*G–I*), adding support to the claim that individual differences in adaptation rate can be explained from differences in noise according to an optimal learning rule. To assess the robustness of our findings, we performed a sensitivity analysis for the model priors (see Table 1: alternative priors 1–3) and a control analysis for within-subject correlations (see Table 1: permuted samples) and found consistent results.

**Table 1.** Sensitivity and control analyses.

	$\sigma_{\eta }\,[s]\;(\beta )$	${\sigma_{\;}}_{\epsilon }\,\;[s]\;(\beta )$	K[s] (r)
Main analysis	0.44 [0.27 0.59]	–0.39 [–0.50 –0.30]	0.54 [0.38 0.66]
Alternative priors 1	0.44 [0.26 0.60]	–0.40 [–0.50 –0.29]	0.53 [0.38 0.66]
Alternative priors 2	0.45 [0.27 0.61]	–0.40 [–0.51 –0.30]	0.53 [0.37 0.66]
Alternative priors 3	0.44 [0.28 0.60]	–0.40 [–0.51 –0.30]	0.53 [0.38 0.66]
Permuted samples	0.29 [0.10 0.45]	–0.38 [–0.50 –0.24]	0.38 [0.21 0.66]

For the main analysis, we used logistic normal distributions with hyperparameters sampled from normal and gamma distributions as priors for A[s] and B[s] and inverse gamma distributions as priors for $\sigma_{\eta }^{2}[s]$ and $\sigma_{\epsilon }^{2}[s]$. For the sensitivity analysis, we used (alternative priors 1) *t*-distributions with the hyperparameter for the degrees of freedom sampled from an exponential distribution as priors for A[s] and B[s] (alternative priors 2) *t*-distributions as priors for A[s] and B[s] and uniform distributions in the range [0,20] as priors for $\sigma_{\eta }$ and $\sigma_{\epsilon }$, and (alternative priors 3) beta distributions with hyperparameters sampled from gamma distributions as priors for A[s] and B[s] and uniform distributions as priors for $\sigma_{\eta }$ and $\sigma_{\epsilon }$. Finally, as a control analysis for within-subjects correlations of the model parameters, we recalculated the correlation and regressions coefficients after permuting the samples of the main analysis differently for each parameter.

Finally, we investigated how planning and execution noise correlated with movement peak velocity. Execution noise originates from muscle activity and should increase with vigorous contraction when larger motor units are recruited which fire at a lower frequency and produce more unfused twitches (Harris and Wolpert, 1998; Jones et al., 2002). Indeed, by regressing peak velocity onto the noise terms, we found a negligible correlation between peak velocity and planning noise *β* = –0.12; 95% HDI = [–0.27 0.02] and a small positive correlation between peak velocity and execution noise *β* = 0.22; 95% HDI = [0.18 0.28].

## Discussion

We investigated the relation between components of motor noise and visuomotor adaptation rate across individuals. If adaptation approximates optimal learning from movement error, it can be predicted from Kalman filter theory that planning noise correlates positively and execution noise negatively with adaptation rate (Kalman, 1960). To test this hypothesis, we performed a visuomotor adaptation experiment in 69 subjects and extracted planning noise, execution noise, and adaptation rate using a state-space model of trial-to-trial behavior. Indeed, we found that adaptation rate correlates positively with planning noise (*β* = 0.44; 95% HDI = [0.27 0.59]) and negatively with execution noise (*β* = –0.39; 95% HDI = [–0.50 –0.30]). In addition, the steady-state Kalman gain calculated from planning and execution noise correlated positively with adaptation rate (*r* = 0.54; 95% HDI = [0.38 0.66]). We discuss implications of our findings for the optimal control model of movement and cerebellar models of adaptation and identify future applications of Bayesian state-space model fitting.

### Optimal control model of movement

The optimal control model of movement has been successful in providing a unified explanation of motor control and motor learning (Todorov and Jordan, 2002). In this framework, the motor system sets a motor goal (possibly in the prefrontal cortex) and judges its value based on expected costs and rewards in the basal ganglia (Shadmehr and Krakauer, 2008). Selected movements are executed in a feedback control loop involving the motor cortex and the muscles which runs on an estimate of the system’s states (Shadmehr and Krakauer, 2008). Both the feedback controller and the state estimator are optimal in a mathematical sense, the feedback controller because it calculates optimal feedback parameters for minimizing motor costs and maximizing performance, given prescribed weighting of these two criteria (Åström and Murray, 2008), and the state estimator because it optimally combines sensory predictions from a forward model (cerebellum) with sensory feedback from the periphery (parietal cortex), similar to a Kalman filter (Kalman, 1960; Wolpert et al., 1995). In the optimal control model of movement, motor adaptation is defined as calibrating the forward model, which is optimal in the same sense as the state estimator (Shadmehr et al., 2010).

Wu et al. (2014) is one of the first studies to suggest that there may be a positive relationship between motor noise and motor adaptation. They outlined two apparent challenges of their findings to the optimal control approach: first, they claimed that optimal motor control is inconsistent with a positive relation between motor noise and adaptation rate; second, they claimed that optimal motor control does not account for the possibility that the motor system shapes motor noise to optimize adaptation. We take a different view. Because we find that only the planning component correlates positively with adaptation rate, our results are predicted by Kalman filter theory (Kalman, 1960) and consistent with optimal control models of movement (Todorov and Jordan, 2002; Åström and Murray, 2008). However, we do agree that the mathematical structure used to express the optimal control approach does not provide a clear way to discuss shaping noise to optimize adaptation. While this may be a technical difficulty from the point of view of optimal feedback approaches, it is apparent that there is electrophysiological evidence that some animals do shape noise to optimize adaptation. This evidence can be found in monkeys (Mandelblat-Cerf et al., 2009). In addition, studies in Bengalese finches show that a basal ganglia-premotor loop learns a melody from reward (Charlesworth et al., 2012) by injecting noise (Kao et al., 2005) to promote exploration (Tumer and Brainard, 2007) during training (Stepanek and Doupe, 2010) and development (Olveczky et al., 2005). We suggest that a similar mechanism operates in humans during adaptation. This additional tuning mechanism could be an interesting topic of future studies into optimal control models of movement.

### Cerebellar model of motor adaptation

Motor adaptation is the learning process which fine tunes the forward model and is believed to take place in the olivocerebellar system (De Zeeuw et al., 2011). How could this learning process be sensitive to planning noise and execution noise on a neuronal level?

Central to the forward model is the cerebellar Purkinje cell, which responds to selected sensory (Chabrol et al., 2015) and motor (Kelly and Strick, 2003) parallel fiber input with a firing pattern reflecting kinematic properties of upcoming movements (Pasalar et al., 2006; Herzfeld et al., 2015). When Purkinje cell predictions of the upcoming kinematic properties are inaccurate, activity of neurons in the cerebellar nuclei is proportional to the prediction error. This is apparently because inhibitory Purkinje cell input cannot cancel the excitatory input from mossy fibers and the inferior olive (Brooks et al., 2015). The sensory prediction error calculated by the cerebellar nuclei could be used to update either (1) motor commands in a feedback loop with (pre)motor areas (Kelly and Strick, 2003) or (2) state estimates of the limb in the parietal cortex (Grafton et al., 1999; Clower et al., 2001). During adaptation, parallel fibers to Purkinje cell synapses associated with predictive signals are strengthened and parallel fibers to Purkinje cell synapses associated with nonpredictive signals are silenced (Dean et al., 2010). These plasticity mechanisms are affected by climbing fibers originating from the inferior olive, which integrate input from the sensorimotor system and the cerebellar nuclei and act as a teaching signal in the olivocerebellar system (De Zeeuw et al., 1998; Ohmae and Medina, 2015).

No previous experimental or modeling work has considered how planning or execution noise might be conveyed to the cerebellum or how they might influence plasticity. We speculate that planning noise is reflected in synaptic variability of the parallel fiber to Purkinje cell synapse. Electrophysiological studies of CA1 hippocampal neurons have shown that synaptic noise can improve detection of weak signals through stochastic resonance (Stacey and Durand, 2000). Such a mechanism might help form appropriate connections at the parallel fiber to Purkinje cell synapse during adaptation. In addition, theoretical studies on deep learning networks have shown that gradient descent algorithms, which can be likened to error-based learning, benefit from adding noise to the gradient at every training step (Neelakantan et al., 2015). Furthermore, we speculate that execution noise affects adaptation through climbing fiber firing modulation. Execution noise will decrease reliability of sensory prediction errors because (1) the motor plan is not executed faithfully (motor noise; van Beers et al., 2004) and (2) the sensory feedback is inaccurate (sensory noise; Osborne et al., 2005). Therefore, when sensory information for a specific movement plan has been unreliable in the past, the olivocerebellar system might decrease its response to sensory prediction error, for example by decreasing climbing fiber firing in the inferior olive (De Zeeuw et al., 1998), which would lower the adaptation rate. The existence of such a mechanism has also been suggested by a recent behavioral study that showed a specific decline in adaptation rate for movement perturbations that had been inconsistent in the past (Herzfeld et al., 2014).

### Two-rate models of adaptation

Our results are based on a one-rate learning model of adaptation (Cheng and Sabes, 2006, 2007; van Beers, 2009). However, recent studies have suggested that a two-rate model composed of a slow but retentive and a fast but forgetting learning system provides a better explanation for learning phenomena such as savings and anterograde interference (Smith et al., 2006). The fast learning system might represent an explicit process, which could be located in the cortex, and the slow learning system an implicit process, which could be located in subcortical areas such as the cerebellum (Mazzoni and Krakauer, 2006; Taylor et al., 2014; McDougle et al., 2015). How could we interpret our results in light of these two-rate models? In a two-rate state-space model, the two systems will add to produce the movement output (Smith et al., 2006). That is, the total adaptation rate is equal to the sum of the adaptation rates of the two systems, and the same goes for the planning noise. Of course, a two-rate model will still include only one term for execution noise. Therefore, a two-rate model can reproduce our results either if both systems are optimally tuned or if only one system is optimally tuned but is relatively dominant. With our current experimental design, we cannot differentiate between these two options. Future studies combining reporting-based approaches to discern the contributions of the implicit and explicit processes and the Bayesian statistical approach to state-space modeling presented in this paper could further unravel this question.

10.1523/ENEURO.0170-18.2018.ed1 Extended data 1 Bugs/Jags code for running the Bayesian state-space model of visuomotor adaptation. Download Extended Data 1, TXT file.

## Synthesis

Reviewing Editor: Trevor Drew, University of Montreal Decisions are customarily a result of the Reviewing Editor and the peer reviewers coming together and discussing their recommendations until a consensus is reached. When revisions are invited, a fact-based synthesis statement explaining their decision and outlining what is needed to prepare a revision will be listed below. The following reviewer(s) agreed to reveal their identity: Reza Shadmehr. Note: If this manuscript was transferred from JNeurosci and a decision was made to accept the manuscript without peer review, a brief statement to this effect will instead be what is listed below.

The authors have addressed all of my previous concerns. I only have some minor comments that they may wish to look at. This is a wonderful paper.

On lines 327 and 329, aren't you reporting r here, not β?

Typo: line 292, should say Figure 4F, not 3F

Typo: line 300, should say Figure 4H, 3H

Typo: line 176: probable, not probably
